# The Role of Milk on Children’s Weight Status: An Epidemiological Study among Preadolescents in Greece

**DOI:** 10.3390/children9071025

**Published:** 2022-07-10

**Authors:** Aikaterini Kanellopoulou, Rena I. Kosti, Venetia Notara, George Antonogeorgos, Andrea Paola Rojas-Gil, Ekaterina N. Kornilaki, Areti Lagiou, Mary Yannakoulia, Demosthenes B. Panagiotakos

**Affiliations:** 1 Department of Nutrition and Dietetics, School of Health Science and Education, Harokopio University, 17671 Athens, Greece; katerkane@gmail.com (A.K.); gantonogeorgos@gmail.com (G.A.); myianna@hua.gr (M.Y.); 2 Department of Nutrition and Dietetics, School of Physical Education, Sports and Dietetics, University of Thessaly, 42132 Trikala, Greece; renakosti@uth.gr; 3 Department of Public and Community Health, Laboratory of Hygiene and Epidemiology, School of Public Health, University of West Attica, 12243 Athens, Greece; venotara@uniwa.gr (V.N.); alagiou@uniwa.gr (A.L.); 4 Department of Nursing, Faculty of Health Sciences, University of Peloponnese, 22100 Tripoli, Greece; apaola71@yahoo.com.mx; 5 Department of Preschool Education, School of Education, University of Crete, 74100 Rethimno, Greece; ekornilaki@edc.uoc.gr; 6 Faculty of Health, University of Canberra, Canberra 2617, Australia

**Keywords:** child, diet, obesity, overweight, milk, public health

## Abstract

Milk consumption contributes greatly to children’s diet, playing a pivotal role in the development and structure of bones and the functioning of the musculoskeletal system and the heart. This study investigated the association between the type of milk and childhood overweight/obesity. In the school period 2014–2016, 1728 students aged 10–12 years and their parents participated. The measurement and classification of their weight status were performed through the criteria of the International Obesity Task Force. Among others, the type of milk consumption was recorded. Four categories of the type of milk children consumed were classified (white milk, chocolate milk, both types of milk, and no milk at all). Children consuming only white milk were 33.1% less likely to be overweight/obese in comparison with children who were not consuming milk at all [OR (95% CI): 0.669 (0.516, 0.867), *p* = 0.002]. The consumption of chocolate milk showed a protective role against childhood overweight/obesity although its association was not consistently significant. This study highlights the significant contribution of milk (and particularly of white milk) consumption to weight management, and thus its promotion should be consistently encouraged. More studies are needed to shed light on the effects of different dairy foods on weight status in childhood.

## 1. Introduction

According to the World Health Organisation, globally, childhood and adolescent obesity have been emerging as a serious problem taking into account that in 2016, 18% of children and youth 5–19 years old were overweight/obese leading to long-term significant adverse health effects in adulthood [1]. Although the intake of dairy products in children’s and adolescents’ diets has been recognised as a marker of better diet quality [2], the majority of youth fail to meet dietary recommendations [3].

Nevertheless, the role of dairy product consumption in children’s and adolescents’ risk of overweight/obesity has attracted scientific interest. So far, several systematic reviews and meta-analyses in the paediatric population have demonstrated inconsistent and conflicting results [4,5,6,7,8,9]. The most recently published German longitudinal study with a median follow-up of 9 years in children demonstrated that the consumption of all types of dairy products was associated with significant increases in Body Mass Index (BMI) (although small) as well as in fat mass and lean mass [10]. The findings of the most recently conducted meta-analyses [9] revealed controversial findings depending on the study design. In particular, from the pooled analysis of cross-sectional studies, it was revealed that total dairy consumption was associated with 34% lower odds of obesity, whereas no significant associations were found between obesity prevalence risk and yoghurt or milk. However, from the pooled analysis of prospective studies in children, due to the limited and inconclusive evidence, it was not feasible to confirm an inverse association [9]. 

Beyond the differences in study design, one plausible explanation behind the observed discrepancies could be attributed to the differences in composition (fat/sugar content), processing type (i.e., fermentation), and form (liquid/solid) of the studied dairy products (i.e., milk, cheese, and yoghurt) [11]. In light of the conflicting evidence, the fact that Greek children have been recognised as among the most obese in Europe, the forecasted increasing trends of dairy product consumption in Greece, as well as the fundamental role of dairies on musculoskeletal health, and the global burden of childhood obesity, the aim of the present study was to investigate the association of the type of milk with weight status through several characteristics in a representative sample of Greek preadolescent students aged 10–12. The research hypothesis was that the type of milk influences the likelihood of obesity.

## 2. Materials and Methods

### 2.1. Design and Setting

An epidemiological, cross-sectional study was conducted in forty-seven schools from five Greek areas as described elsewhere [12].

### 2.2. Sampling Procedure

Sampling randomisation was used for schools’ participation in the study, by a list that the Greek Ministry of Education provided. All students from the two last grades of primary school, were eligible to participate in the research. In the school years 2014–2015 and 2015–2016, a sample of 1728 preadolescents between 10 and 12 years old participated in the study. Among the schools, the participation rate varied between 95 and 100%. For the aims of our study, only students whose type of milk consumption was enrolled were analysed; therefore, the final working sample was 1710 children.

### 2.3. Measurements

A specially designed research tool was completed anonymously by students. The questionnaire included demographic characteristics (sex, age, place of residence), several other characteristics such as dietary habits using semi-quantitative Food Frequency Questionnaire [13], and physical activity (using the Lifestyle Questionnaire (PALQ)) [14]. 

Anthropometric characteristics of students, i.e., height and weight, were measured, according to instructions, by specially trained health researchers as described in other studies [12,15]. The calculation of students’ BMI was classified according to the International Obesity Task Force (IOTF) BMI cut-off criteria [16] as either with normal weight or with overweight/obesity. Due to the low frequency of children with obesity, we grouped together the children with overweight and obesity. 

Moreover, a validated tool was used for the evaluation of students’ adherence to the Mediterranean diet (KIDMED Score) [17]. For more information, the interested reader is referred to [18]. 

The type of milk that children consumed (i.e., whole white, low-fat milk, fat-free milk, or/and chocolate milk), its consumption frequency, and the cheese and yoghurt consumption frequency were also recorded, through the validated PANACEA study Food Frequency Questionnaire [13]. To assess milk consumption, we created a new variable with four values: 1 if children consumed only white milk, 2 if children consumed only chocolate milk, 3 if children consumed both types of milk, and 4 if children did not drink milk. Due to the small number of children consuming low-fat milk and/or fat-free milk, these types of milk were not included in the new variable. Moreover, the frequency of breakfast and meal consumption was recorded. All frequencies of consumption were adjusted on a weekly basis.

Furthermore, items about several characteristics of parents, i.e., socio-demographic characteristics (age, educational level, income, place of residence), marital status (married/ single parents/divorced/widowed), were completed by children’s parents, as described elsewhere [15]. 

### 2.4. Statistical Power Analysis

The command *power* of the Stata 14.0 software (M. Psarros & Assoc., Sparti, Greece) was used for the a priori sample size calculation, to achieve statistical power >80%, as described in previous publications [12,15,18,19].

### 2.5. Bioethics

The research was carried out following the principles of the 1989 Declaration of Helsinki of the World Medical Association [20]. The approval of the research was requested from the appropriate department of the Ministry of Education and Religious Affairs (code F15/396/72005/C1) by the Institute of Educational Policy of the Ministry of Education and Religious Affairs. The investigators informed all individuals who were involved about the purposes and procedures of the research. Furthermore, the parents allowed their children to participate in the study by written consent.

### 2.6. Statistical Analysis

Standard deviation (SD) and mean were used for continuous variables. Relative and absolute frequencies (percentages) were used for categorical variables. To assess the effect of milk consumption on the students’ weight status, eight nested logistic regression models were applied using BMI categories (children with normal weight vs. children with overweight/obesity) as the dependent variable and the milk variable as the independent one, adjusting for age, sex, KIDMED score, type of residence, type of family structure, and educational level of parents. By odds ratio (OR) and 95% Confidence Intervals (CI) are presented all logistic regression results. Every statistical test was two-sided, and the level of significance was set at 5%. For the statistical analyses Stata 14.0 (M. Psarros & Assoc., Sparti, Greece) was used.

## 3. Results

### 3.1. Prevalence of Overweight/Obesity

The total prevalence of overweight/obesity was 27.5%. Sex-specific analysis revealed that 32.5% of boys and 23.4% of girls were categorised in the group of children with overweight/obesity (*p* < 0.001 for sex difference). 

Children’s socio-demographic characteristics and lifestyle habits by body weight status are presented in Table 1. Boys were more likely to be overweight/obese compared to girls, children engaged in physical activities were less likely to be overweight/obese compared to those who followed sedentary behaviours, children whose parents were higher educated were less likely to be overweight/obese compared to those whose parents had basic/secondary education, and children from Athens were less likely to be overweight/obese compared to children from other areas of Greece. Moreover, children with overweight/obesity had significantly higher adherence to the Mediterranean diet than children with normal weight.

Children’s lifestyle habits and socio-demographic characteristics, by type of milk, are presented in Table 2. The type of milk that children consume was significantly associated with sex (*p* < 0.001), physical activity (*p* = 0.004), KIDMED Score (*p* < 0.001), weekly milk consumption frequency (*p* = 0.003), and daily meals frequency (*p* < 0.001). More specifically, boys preferred to drink chocolate milk or both types of milk, while girls preferred to drink white milk or not drink any milk at all. Similarly, children who exercised preferred to drink white milk or not drink any milk at all. Moreover, children with higher values of KIDMED score preferred to drink white milk or not drink any milk at all. Children who consumed milk more frequently per week preferred to drink white milk, while children who consumed milk less frequently preferred to drink chocolate milk. Finally, the more meals children had per day, the more white milk they preferred to drink.

### 3.2. Children’s Weight Status and Its Association with Milk Consumption

In Table 3, the crude associations between the type of milk that children consume and their weight status are presented. Children consuming only white milk were 30.4% less likely to be overweight/obese compared to those who were not consuming white milk. Although not significant, children consuming only chocolate milk were 14.5% more likely to be overweight/obese compared to those who were not consuming chocolate. Children consuming only white milk were 33.1% less likely to be overweight/obese compared to those who were not consuming milk at all. Although not significant, children consuming milk of either type less than two times per week were 19% more likely to be overweight/obese compared to those who consumed milk more than two times per week.

The weekly milk consumption per several children’s characteristics is shown in Table 4. Children from families with a higher income consumed more milk per week than children from families with less income (5.43 ± 2.442 vs. 5.74 ± 2.207, *p* = 0.027). Similarly, children who consumed breakfast five or more times per week consumed more milk per week than children who consumed breakfast less than five times per week (4.68 ± 2.725 vs. 6.01 ± 1.991, *p* < 0.001). Moreover, the more meals children had per day, the more milk they consumed per week (4.98 ± 2.687 for children with 1–2 meals per day, 5.38 ± 2.408 for children with 3 meals per day, and 5.94 ± 2.089 for children with more than 3 meals per day, *p* < 0.001). 

Weekly milk consumption had a low positive correlation with KIDMED score (Pearson’s r = 0.183, *p* < 0.001) and health literacy (Pearson’s r = 0.181, *p* < 0.001). Moreover, children consumed milk more often per week than their parents (5.56 ± 2.35 vs. 3.91 ± 2.99, *p* < 0.001). 

### 3.3. Association between Type of Milk and Children’s Weight Status–Multivariate Analysis

However, residual confounding may exist due to the observational nature of this study. In Table 5, the results from nested logistic regression models evaluating the association between milk consumption and children’s weight status, after taking into consideration various children’s characteristics, are presented. In total, eight models were estimated. As it can be drawn from model 1, children consuming white milk were 33.1% less likely to be overweight/obese compared to children consuming no milk at all. No such association was revealed comparing children consuming chocolate milk to those not consuming milk and children consuming both types of milk to those not consuming milk. This association, between milk consumption and weight status, remained significant irrespective of sex, age, and physical activity level of children (models 2 and 3). However, when adherence to the Mediterranean diet was taken into account (model 4), it was observed that also the effect of chocolate milk consumption was significant at the 10% significant level; children consuming chocolate milk were 28.0% less likely to be overweight/obese compared to children consuming no milk at all. When the milk consumption frequency was taken into account (model 5), it was observed that also the effect of the consumption of both types of milk was significant at the 10% significant level; children consuming both types of milk were 35.7% less likely to be overweight/obese compared to children consuming no milk. Taking into account milk consumption frequency (parents) (model 6), mother’s and father’s educational level (model 7), and family structure and residence (model 8), the effect of both types of milk lost its significance, but the effect of consuming chocolate milk was enhanced. In all eight models, the effect of consuming white milk was consistently significant.

## 4. Discussion

In the present research, the association between milk type consumption and preadolescents’ weight status was investigated. Findings showed that the consumption of white milk is consistently and inversely associated with children’s weight status following the adjustment of several confounding factors. Moreover, the consumption of chocolate milk showed a protective role against childhood overweight/obesity, although its association was not always significant. On the other hand, no association was found between cheese and yoghurt consumption with weight status. Despite the limitations of the present study due to its cross-sectional design, our results convey interesting messages from a public health perspective shedding more light on the understanding of modifiable risk factors of preadolescents’ overweight/obesity which are considered crucial for preventing the risk of obesity. The importance of the investigation of the effect of milk consumption on weight status in Greece is further supported by the increasing trends of dairy product consumption in the last decade in both the milk and yogurt sectors (https://www.statista.com/outlook/cmo/food/dairy-products-eggs/greece#volume, accessed on 5 July 2022).

Consistent with the literature [21], our findings confirmed that milk consumption is influenced by several socio-demographic (i.e., family income) and lifestyle factors (i.e., physical activity and diet quality) in adolescents, with males o consuming more milk compared to females. Our findings are in accordance with the results of another cross-sectional study in children which showed that milk consumption was negatively associated with BMI [22]. A meta-analysis of observational studies [6] concluded that dairy products, and in particular milk consumption, was associated with a decreased risk of obesity [0.81 (0.75–0.88)] in children [6]. In agreement with our findings, the conclusions of an umbrella review in children and adults demonstrate that milk intake was inversely associated with obesity. Moreover, the findings of another cross-sectional study conducted in adolescents showed that milk intake was inversely associated with BMI and only in girls. The authors concluded that no association was found as regards cheese and yoghurt intake with BMI [23], which is in line with the findings of the present study. 

In the present study, milk consumption, and in particular white milk compared to chocolate milk, consistently showed an inverse association with BMI. There is a lot of scepticism as regards the contribution of added sugars in flavoured milk in diet quality and its role in the prevalence of overweight/obesity [24,25]. However, despite its added sugar content [26], flavoured milk has a nutrient profile similar to that of white milk, outweighing the detriments of added sugar [24]. Findings from a longitudinal study showed that flavoured milk’s consumption, although with less favourable changes in body fat compared to non-consumers, is not related to weight gain, which is in agreement with our findings [27]. On the other hand, cheese and yoghurt showed no association with weight status. 

Some plausible explanations are that, in adolescence, the consumption of milk per se could be considered as a marker of healthier dietary habits either through the partial substitution of unhealthy habits such as sugar-sweetened beverage intake [26] or through the enhancement of other healthy habits, for instance, breakfast consumption [28,29,30]. The literature suggests that milk consumption seems to have a protective effect against overweight/obesity, irrespective of yoghurt or cheese consumption [31]. 

In the present study, the diversified findings of the effects of milk, yoghurt, and cheese on weight status are further supported by the literature suggesting the distinct characteristics of the food matrices of dairy products on health outcomes [32]. The nutritive value of dairy products has been well recognised in the literature, underscoring that dairy products are a rich source of high-quality protein and multiple essential micronutrients such as magnesium, zinc, calcium, phosphorus, potassium, and vitamins A, B2, B12, and D [3]. The potential beneficial effects of dairy foods on weight status have not yet been fully elucidated and could be attributed to several factors. Both protein and calcium have been linked to weight status and body composition. Calcium may contribute to weight regulation by decreasing de novo lipogenesis, increasing lipolysis [33], or interfering with fat absorption in the intestine [34], causing a decrement in energy intake. Moreover, dairy protein has been demonstrated to reduce appetite [35], as well as to regulate body composition by diet-induced thermogenesis, increasing satiety, decreasing hunger, and preserving or increasing lean mass [36,37]. Milk provides a range of bioactive peptides which have been demonstrated to exert an inhibitory effect on angiotensin-converting enzyme, resulting in the inhibition of fat deposition through intermediate pathways [38]. In addition, many fermented foods such as yoghurt and cheese also contain probiotics which have been studied for their potential beneficial health effects on microbiota [39]. Recent evidence suggests that the difference in the fat content across the range of dairy products (i.e., skim, low fat, or whole milk) does not seem to be really an issue of concern [40], but rather the type of dairy product in terms of its food matrix may be more important for preventing long-term weight gain [41]. The weight of evidence suggests that dairy foods do not promote weight gain [19], but rather regulate body composition by reducing body fat and increasing muscle mass [5,41]. 

The results of our study indicate that milk consumption plays a significant role in weight management. To further promote milk consumption, actions should be taken either by the government or other institutions. To this end, the EU School fruit, vegetables, and milk scheme is implemented, in which more than 800 Greek schools participate (https://ec.europa.eu/info/food-farming-fisheries/key-policies/common-agricultural-policy/market-measures/school-fruit-vegetables-and-milk-scheme/country/greece_en, accessed on 5 July 2022). Moreover, the DIATROFI Programme (http://diatrofi.prolepsis.gr/en, accessed on 5 July 2022), a school feeding program, has provided meals to approximately 113,000 students over the last 10 years.

However, there are some limitations that should be considered, given that this study has a cross-sectional design. Firstly, causal inference and temporal relationship cannot be established. No information about chronic medical problems or any severe food allergies affecting food intake and dietary restrictions involving animal products were gathered. Moreover, the results cannot be generalised to all Greek children, but only to those of this age range (i.e., 10–12 years old). The participating children came from specific areas and not from all over Greece. However, the large sample size and the implemented stratified random sampling scheme increase the representativeness of the sample. Because self-reporting questionnaires were used, reporting bias may also be considered as a potential limitation. In our opinion, children, because of their childhood innocence, gave honest and truthful answers. However, the presence of a trained investigator throughout the completion of the questionnaire increases the validity of the responses. For this reason, the children’s responses were not verified by parents.

## 5. Conclusions

The observed discrepancies on the weight status across the different types of dairy products make firm conclusions difficult. Nevertheless, the present study highlights the significant contribution of milk, and in particular of white milk, consumption in weight management, and thus its promotion should be consistently encouraged. Further research on the effects of the different dairy foods on weight status/adiposity is warranted through longitudinal studies. 

## Figures and Tables

**Table 1:** Children’s socio-demographic characteristics and lifestyle habits, by body weight status.

Children’s Characteristics	Total Population (*n* = 1710)	Normal Weight Children ** (*n* = 1220)	Children with Overweight/Obesity ** (*n* = 464)	*p* *
Age	11.21 (0.79)	11.23 (0.79)	11.15 (0.78)	0.06
Sex				
Boys	783 (45.8%)	520 (42.6%)	250 (53.9%)	<0.001
Girls	927 (54.2%)	700 (57.4%)	214 (46.1%)	
Physical activity (yes)	1254 (78.6%)	915 (80.8%)	319 (73.0%)	<0.001
KIDMED Score (−4 to 12)	4.62 (2.30)	4.70 (2.28)	4.39 (2.33)	0.01
Cheese (times/week)	3.44 (2.66)	3.47 (2.66)	3.34 (2.65)	0.38
Yoghurt (times/week)	2.27 (2.40)	2.27 (2.36)	2.27 (2.48)	0.99
Mother’s educational level				
Basic-secondary	633 (55.2%)	447 (52.8%)	176 (62.0%)	0.007
Higher	514 (44.8%)	400 (47.2%)	108 (38.0%)	
Father’s educational level				
Basic-secondary	688 (60.1%)	482 (57.2%)	193 (67.7%)	0.002
Higher	457 (39.9%)	361 (42.8%)	92 (32.3%)	
Residence				
Athens	1107 (66.9%)	812 (68.6%)	275 (61.2%)	0.005
Other	547 (33.1%)	372 (31.4%)	174 (38.8%)	
Marital Status				
Married	1023 (88.6%)	759 (89.5%)	251 (87.2%)	0.27
Non-married	131 (11.4%)	89 (10.5%)	37 (12.8%)	
Family income				
≦18,000	539 (49.4%)	386 (48.1%)	142 (52.4%)	0.22
>18,000	551 (50.6%)	417 (51.9%)	129 (47.6%)	
Weekly breakfast consumption				
Less than 5 times	507 (29.8%)	353 (29.0%)	145 (31.5%)	0.32
5 times or more	1196 (70.2%)	864 (71.0%)	315 (68.5%)	
Daily meals frequency				
1–2 times	228 (13.8%)	164 (13.8%)	62 (14.1%)	0.84
3 times	600 (36.2%)	431 (36.2%)	160 (36.4%)	
More than 3 times	828 (50.0%)	596 (50.0%)	218 (49.5%)	

Children’s eating habits are presented as mean (SD) and categorical variables as frequencies (%), * Level of significance set at *p* < 0.05; tested via independent samples *t*-test for eating habits, and chi-square test for all other categorical variables ** Weight status is defined based on BMI cut-offs for adults and on IOTF cut-off criteria for children. SD = standard deviation; IOTF = international obesity task force; BMI = body mass index.

**Table 2:** Children’s socio-demographic characteristics and lifestyle habits, by milk consumption characteristics (type of milk).

Children’s Characteristics	Whole White (*n* = 979)	Chocolate (*n* = 247)	Both (*n* = 100)	Neither (*n* = 384)	*p* *
Age	11.17 (0.79)	11.27 (0.77)	11.36 (0.70)	11.23 (0.81)	0.05
Sex					
Boys	445 (45.5%)	132 (53.4%)	56 (56.0%)	150 (39.1%)	<0.001
Girls	534 (54.5%)	115 (46.6%)	44 (44.0%)	234 (60.9%)	
Physical activity (yes)	721 (79.1%)	164 (71.0%)	71 (75.5%)	298 (83.2%)	0.004
KIDMED Score (−4 to 12)	4.81 (2.32)	3.94 (2.44)	4.31 (2.30)	4.67 (2.05)	<0.001
Milk (times/week)	5.78 (2.14)	5.24 (2.50)	5.64 (2.33)	5.43 (2.58)	0.003
Cheese (times/week)	3.37 (2.61)	3.24 (2.70)	3.49 (2.86)	3.72 (2.70)	0.10
Yoghurt (times/week)	2.30 (2.36)	2.08 (2.49)	2.29 (2.47)	2.31 (2.43)	0.62
Mother’s educational level					
Basic-secondary	367 (57.1%)	100 (58.8%)	28 (49.1%)	138 (49.8%)	0.12
Higher	276 (42.9%)	70 (41.2%)	29 (50.9%)	139 (50.2%)	
Father’s educational level					
Basic-secondary	394 (61.1%)	105 (61.4%)	37 (64.9%)	152 (55.9%)	0.40
Higher	251 (38.9%)	66 (38.6%)	20 (35.1%)	120 (44.1%)	
Residence					
Athens	625 (65.9%)	171 (71.3%)	59 (62.8%)	252 (67.7%)	0.35
Other	323 (34.1%)	69 (28.7%)	35 (37.2%)	120 (32.3%)	
Family marital status					
Married	578 (89.5%)	150 (85.7%)	53 (93.0%)	242 (87.7%)	0.35
Divorced/separated	68 (10.5%)	25 (14.3%)	4 (7.0%)	34 (12.3%)	
Family income					
≦18,000	301 (50.2%)	87 (53.0%)	30 (53.6%)	121 (44.8%)	0.20
>18,000	299 (49.8%)	77 (47.0%)	26 (46.4%)	149 (55.2%)	
Weekly breakfast consumption					
Less than 5 times	270 (27.7%)	93 (37.7%)	39 (39.4%)	105 (27.4%)	0.62
5 times or more	704 (72.3%)	154 (62.3%)	60 (60.6%)	278 (72.6%)	
Daily meals frequency					
1–2 times	140 (14.8%)	35 (14.7%)	18 (18.4%)	35 (9.4%)	<0.001
3 times	364 (38.4%)	80 (33.6%)	31 (31.6%)	125 (33.6%)	
More than 3 times	444 (46.8%)	123 (51.7%)	49 (50.0%)	212 (57.0%)	

Children’s eating habits are presented as mean (SD) and categorical variables as frequencies (%), * Level of significance set at *p* < 0.05; tested via independent samples *t*-test for eating habits, and chi-square test for all other categorical variables, SD = standard deviation.

**Table 3:** The association between type of milk and weight status.

		Normal Weight Children (*n* = 1220)	Children with Overweight/Obesity (*n* = 464)	OR ^§^ (95% CI)
Whole white milk	Yes	799 (65.5%)	264 (56.9%)	0.696 (0.559, 0.865)
	No	421 (34.5%)	200 (43.1%)	
Chocolate milk	Yes	241 (19.8%)	102 (22.0%)	1.145 (0.882, 1.486)
	No	979 (80.2%)	362 (78.0%)	
Milk (times/week)		5.63 (2.32)	5.62 (2.28)	NA
Milk	Whole white	726 (59.5%)	238 (51.3%)	0.669 ^†^ (0.516, 0.867)
	Chocolate	168 (13.8%)	76 (16.4%)	0.923 ^†^ (0.653, 1.304)
	Both	73 (6.0%)	26 (5.6%)	0.727 ^†^ (0.442, 1.194)
	Neither	253 (20.7%)	124 (26.7%)	–
Weekly milk consumption	Less than two times	138 (11.4%)	45 (9.8%)	1.190 (0.834, 1.697)
	Two times or more	1072 (88.6%)	416 (90.2%)	

OR, odds ratio, 95% CI, 95% confidence interval. § OR to be overweight/obese. † OR computed with “Neither” as reference category. NA, not applicable.

**Table 4:** Weekly milk consumption per several children’s characteristics.

		N	Mean ± SD	*p* *
Sex	Boys	778	5.67 ± 2.274	0.38
	Girls	918	5.57 ± 2.347	
Physical activity	No	338	5.42 ± 2.434	0.06
	Yes	1247	5.70 ± 2.248	
Mother’s educational level	Basic-secondary	628	5.52 ± 2.370	0.48
	Higher	511	5.62 ± 2.327	
Father’s educational level	Basic-secondary	682	5.47 ± 2.432	0.05
	Higher	455	5.74 ± 2.192	
Family marital status	Married	1015	5.62 ± 2.306	0.16
	Divorced/separated	130	5.29 ± 2.556	
Residence	Athens	1100	5.57 ± 2.339	0.07
	Other	542	5.79 ± 2.204	
Family income	≦18,000	534	5.43 ± 2.442	0.027
	>18,000	547	5.74 ± 2.207	
Breakfast consumption (times/week)	Less than 5 times	502	4.68 ± 2.725	<0.001
	5 times or more	1188	6.01 ± 1.991	
Daily meals frequency	1–2 times	227	4.98 ± 2.687	<0.001
	3 times	595	5.38 ± 2.408	
	More than 3 times	823	5.94 ± 2.089	

* Level of significance set at *p* < 0.05.

**Table 5:** The association between milk consumption and children’s weight status category (overweight/obesity vs. normal), adjusted for several characteristics through nested logistic regression models (OR, 95% CI).

	Model 1	Model 2	Model 3	Model 4	Model 5	Model 6	Model 7	Model 8
Milk type								
Whole white	0.669 ** (0.516; 0.867)	0.640 *** (0.492; 0.832)	0.638 ** (0.486; 0.839)	0.64 2 ** (0.489; 0.845)	0.632 ** (0.480; 0.833)	0.687 ** (0.488; 0.965)	0.617 ** (0.435; 0.874)	0.618 ** (0.431; 0.862)
Chocolate	0.923 (0.653; 1.304)	0.866 (0.610; 1.229)	0.749 (0.518; 1.084)	0.720 * (0.497; 1.045)	0.705 * (0.484; 1.025)	0.593 ** (0.370; 0.950)	0.546 ** (0.336; 0.887)	0.521 ** (0.315; 0.862)
Both	0.727 (0.442; 1.194)	0.679 (0.411; 1.122)	0.663 (0.395; 1.113)	0.650 (0.387; 1.093)	0.643* (0.382; 1.083)	0.963 (0.499; 1.860)	0.913 (0.459; 1.815)	0.924 (0.451; 1.890)
Neither (ref)	-	-	-	-	-	-	-	-
Sex (boys vs. girls)	-	1.596 *** (1.284; 1.983)	1.654 *** (1.319; 2.075)	1.654 *** (1.319; 2.075)	1.614 *** (1.286; 2.026)	1.780 *** (1.337; 2.371)	1.788 *** (1.330; 2.403)	1.893 *** (1.392; 2.573)
Age (per 1 year increase)	-	0.872 * (0.760; 1.001)	0.837 ** (0.725; 0.967)	0.831 ** (0.719; 0.960)	0.832 ** (0.720; 0.963)	0.809 ** (0.678; 0.966)	0.811 * (0.677; 0.973)	0.820 ** (0.680; 0.989)
Physical activity (yes vs. no)	-	-	1.703 *** (1.307; 2.220)	1.667 *** (1.277; 2.174)	1.677 *** (1.284; 2.190)	1.814 *** (1.290; 2.549)	1.720 ** (1.210; 2.447)	1.842 *** (1.281; 2.649)
KIDMED Score (per 1 unit increase)	-	-	-	0.944 ** (0.898; 0.992)	0.940 ** (0.893; 0.989)	0.946 * (0.886; 1.010)	0.947 (0.885; 1.013)	0.938 * (0.875; 1.006)
Milk consumption frequency (children) (per 1 time increase)	-	-	-	-	1.027 (0.977; 1.080)	0.972 (0.914; 1.033)	0.982 (0.922; 1.046)	0.980 (0.919; 1.046)
Milk consumption frequency (parents) (per 1 time increase)	-	-	-	-	-	1.040 (0.991; 1.090)	1.044 * (0.994; 1.096)	1.046 * (0.994; 1.101)
Mother’s educational level (higher vs. basic-secondary)	-	-	-	-	-	-	0.793 (0.572; 1.099)	0.800 (0.570; 1.122)
Father’s educational level (higher vs. basic-secondary)	-	-	-	-	-	-	0.711 ** (0.507; 0.096)	0.671 ** (0.473; 0.951)
Family structure (nuclear vs. single-parent)	-	-	-	-	-	-	-	0.890 (0.618; 1.283)
Residence (Athens vs. other)	-	-	-	-	-	-	-	0.979 (0.604; 1.588)

OR, odds ratio; 95% CI, 95% confidence interval. *** *p* < 0.001; ** *p* < 0.05; * *p* < 0.10.

## Data Availability

The data presented in this study are available on request from the corresponding author. The data are not publicly available due to privacy and ethical reasons.
